# Sex-related differences in outcome of thoracic aortic surgery

**DOI:** 10.1186/s13019-024-02735-6

**Published:** 2024-04-16

**Authors:** Hongxue Jiang, Hongjie Xu, Zhiyun Xu

**Affiliations:** 1 Department of Gastroenterology, Beijing Friendship Hospital, Capital Medical University, Beijing, China; 2 Department of Cardiovascular Surgery, Changhai Hospital, Second Military Medical University, 168 Changhai Road, Shanghai, 200168 China

**Keywords:** Thoracic aortic aneurysms, Thoracic aortic surgery, Gender, Risk factors, Mortality

## Abstract

**Background:**

Sex-related dissimilarities’ influence on outcomes following thoracic aortic surgery is poorly understood. Our aim is to examine sex-related disparities in patients undergoing thoracic aortic aneurysm (TAA).

**Methods:**

A total of 455 cases undergoing thoracic aortic aneurysm (TAA) surgery were consecutively enrolled between December 2009 and December 2015 in a Chinese hospital. Primary outcomes, including overall mortality and related risk factors, were evaluated. Cox regression is utilized to recognize the independent risk factor of these consequences.

**Results:**

Females, compared to males, had greater indexed aortic diameters and higher aortic transvalvular pressure differences. For the location of aortic aneurysms, females had a higher rate of aortic arch involvement, while males had a higher rate of root involvement. Females underwent less frequent complex proximal aortic operations compared with males (29.5% versus 46.9%; *p* < 0.001). Women and men both had a lower rate of aortic transvalvular pressure difference and LV volume index 7 days after thoracic aortic surgery. The overall mortality for the women’s groups (11%) was suggestively greater compared to 4.9% for the men’s groups (*p* = 0.026). Renal failure and aortic arch involvement were the main risk factors associated with males’ survival, while maximum indexed aortic diameter and cross-clamp time were the risk factors associated with females’ survival.

**Conclusions:**

The outcome after TAA surgery was less favorable in women with significantly increased overall mortality. It highlights the need to focus on implementing personalized surgery strategies and gender-specific guidelines in treating female patients following TAA surgery.

## Background

Women and men have discrepancies in the occurrence, therapy, and outcome of cardiovascular illness [1]. In recent years, gender-related dissimilarities have been determined in patients undergoing a variety of surgeries, including coronary surgery, mitral valve surgery, coronary surgery, united valve, and ruptured abdominal aortic aneurysm surgery [2]. As a common serious disease, thoracic aortic aneurysm (TAA) requires surgical intervention because of the threat of dissection or rupture [3]. The surgical treatment of a TAA is frequently performed in the world. The aorta can be repaired endovascularly or replaced by a synthetic tube (graft) in the thorax or by open surgery (TEVAR) [4], here TEVAR standed for thoracic endovascular aortic repair, which was a minimally invasive procedure used to treat thoracic aortic aneurysms. While brain/or heart damage derived from a variety of factors such as atheromatous or calcium plaques, reduced circulatory blood flow, long circulatory arrest time, and cardiac dysfunction [5, 6] are very common during or after surgery. To date, TAA surgery has a mortality rate up to 10% [7]. There is a need to improve the perioperative outcomes of patients undergoing TAA surgery [8]. A personalized approach to patients undergoing TAA surgery may prove to be an effective policy to improve total outcomes.

Given the physiological, genetic, behavioral, and psychological dissimilarities between women and men, there is the possibility of differences in surgical outcomes. We conducted this study to investigate gender dissilimarities in patients undergoing TAA surgery and assess the sex-related differences in clinical outcomes.

## Methods

### Study population

Between December 2009 and December 2015, patients (*n* = 455) with thoracic aortic aneurysms undergoing thoracic aortic surgery were retrospectively enrolled at the Department of Cardiovascular Surgery of Changhai Hospital (Shanghai, China). Inclusion criteria of patients undergoing TAA surgery (median sternotomy) include total arch reconstructions, hemiarch replacements, descending thoracic aortic repairs, and distal arch. Thoracic-abdominal aortic repair cases requiring circulatory arrest or cases not requiring aortic repairs such as congenital conditions and tumor excision, and emerging cases were excluded from this study. The medical records of the patients, including demographics, clinical findings, the sex difference in the outcome, and follow-up, were collected. The Institutional Review Committee approved this research and all informed contents were obtained.

### Outcome measure

The primary outcomes for evaluation included locations of thoracic aortic aneurysms, baseline Characteristics of patients before surgery and 7 days after thoracic aortic surgery, in-hospital mortality, overall mortality, and major morbidity and risk factors for overall mortality.

### Statistical methods

Categorical data were expressed as a percentage (%), and continuous variables were expressed as mean ± standard deviation. Student *t*-test was utilized for comparing continuous variables. The nominal variable was compared using *Chi*-square or *Fisher’s exact* tests. Cox regression was used to describe appropriate risk factors for overall death. A *p*-value of fewer than 0.05 (two-tailed) was considered statistically significant. Statistical analysis was executed by SPSS 21.0 software (Chicago, SPSS, IL).

## Results

### Baseline characteristics

Of 455 patients, there were 309 (67.9%) men and 146 (32.1%) women. Male and female patients had similar ages (*P* = 0.843), whereas male patients had a higher ratio of coronary artery illness (*P* = 0.023), deprived ventricular functions (*P* = 0.020), and previous cardiac surgery (*P* = 0.004).

Other medical histories, including the incidence of hypertension *(P* = 0.656), diabetes mellitus (*P* = 0.192), dyslipidemia (*P* = 1.000), and renal insufficiency (*P* = 0.513) were not different between the two groups. Anatomically, female patients had a smaller body surface region (*P* < 0.001), translating into higher indexed aortic diameters (*P* = 0.001). For valve anatomy, both male and female patients had a similar proportion of bicuspid aortic valve (*P* = 0.250) and aortic valve stenosis (*P* = 0.089), whereas female patients had a higher aortic valve transvalvular pressure difference (*P* = 0.036), and male patients had a superior ratio of aortic valve insufficiency (*P* < 0.001). The differences in baseline characteristics between women and men were summarized in Table 1.

**Table 1:** Baseline characteristics

Variables	Overall (*n* = 455)(%)	Female (*n* = 146)(%)	Male (*n* = 309((%)	*P* Values
Age, y	54.6 ± 12.72	54.5 ± 12.39	54.7 ± 12.90	0.843
Body surface area, m2	1.8 ± 0.17	1.6 ± 0.14	1.8 ± 0.15	<0.001
Maximum aortic diameter (mm/m2)	51.1 ± 9.58	49.9 ± 9.75	51.7 ± 9.45	0.055
Maximum indexed aortic diameter, mm/m2	29.1 ± 6.00	30.5 ± 6.34	28.5 ± 5.73	0.001
Marfan´s syndrome (%)	19(4.2)	10(6.8)	9(2.9)	0.076
Hypertension(%)	153(33.6)	47(32.2)	106(34.3)	0.656
Diabetes mellitus(%)	17(3.7)	8(5.5)	9(2.9)	0.192
Dyslipidemia(%)	25(5.5)	8(5.5)	17(5.5)	1.000
Renal insufficiency	10(2.2)	2(1.4)	8(2.6)	0.513
Smoking(%)	119(26.2)	2(1.4)	117(37.9)	<0.001
Previous Cardiac Surgery(%)	41(9.0)	5(3.4)	36(11.7)	0.004
Coronary artery disease(%)	34(7.5)	5(3.4)	29(9.4)	0.023
Left ventricular ejection fraction(%)				0.020
LVEF > 60%	222(48.8)	85(58.2)	137(44.3)	
LVEF 40–60%	215(47.2)	57(39.1)	158(51.1)	
LVEF 20–40%	18(4.0)	4(2.7)	14(4.6)	
LVEF < 20%	0(0)	0(0)	0(0)	
NYHA class(%)				0.868
I	11(2.4)	3(2.1)	8(2.6)	
II	186(40.9)	59(40.4)	127(41.1)	
III	238(52.3)	79(54.1)	159(51.4)	
IV	20(4.4)	5(3.4)	15(4.9)	
Aortic valve anatomy (%)				0.250
Tricuspid valve	378(83.1)	117(80.1)	261(84.5)	
Bicuspid valve	77(16.9)	29(19.9)	48(15.5)	
Aortic stenosis(%)	189(41.5)	69(47.3)	120(38.8)	0.089
Aortic insufficiency(%)	330(72.5%)	90(61.6%)	240(77.7%)	<0.001
Aortic valve transvalvular pressure difference	57.1 ± 45.55	72.8 ± 52.62	52.1 ± 39.44	0.036

LVEF: left ventricular ejection fraction. A *p* value < 0.05 was considered statistically significantt

Moreover, there were significant differences in terms of the proportion of the location of aortic aneurysms, including aortic root, aortic arch, ascending aorta, and aortic root between female and male patients (see Table 2). In brief, Female patients had a higher rate of arch involvement (*P* = 0.005), whereas the male group had a greater rate of root involvement (*P* = 0.003). In addition, male patients had a higher proportion of aortic aneurysms involving ascending aorta and aortic root (*P* = 0.001) and arch or/and descending aortic involvement (*P* = 0.034) compared to female patients (see Table 2).

**Table 2:** Location differences of thoracic aortic aneurysms between female and male patients

Variables	Overall (*n* = 455)(%)	Female (*n* = 146)(%)	Male (*n* = 309)(%)	*P* Values
Location (%)				
Aortic root	75(16.5)	13(8.9)	62(20.1)	0.003
Ascending aorta	416(91.4)	136(93.2)	280(90.6)	0.367
Aortic arch	35(7.7)	19(13.0)	16(5.2)	0.005
descending aorta	18(4.0)	8(5.5)	10(3.2)	0.303
Ascending aorta and aortic root involvement	40(8.8)	4(2.7)	36(11.7)	0.001
Arch or/and descending aortic involvement	40(8.8)	19(13)	21(6.8)	0.034

A *p* value < 0.05 was considered statistically significant

### Operative features

Female patients experienced fewer frequent multifaceted proximal aortic operations, including the Bentall procedure (26% versus 40.5%; *P* = 0.003). The rate of the procedure of arch replacement, including Hemiarch replacement (*P* = 0.092), overall arch replacement (*P* = 0.115), and elephant trunk repair (*P* = 1.000), was similar between the two groups, as was the rate of concomitant surgeries (32.2% versus 36.5%; *P* = 0.361). There was no significant dissimilarity in the cardiopulmonary bypass periods (*P* = 0.362), cross-clamp times (*P* = 0.367), and circulatory arrest periods (*P* = 0.068). Female patients had a higher intraoperative transfusion rate (78.1% versus 61.5%; *P* < 0.001), and this was driven by the utilization of fresh frozen plasma and packed red blood cells (see Table 3).

**Table 3:** Intraoperative differences between female and male patients

Variable	Overall (*n* = 455)(%)	Females (*n* = 146)(%)	Males (*n* = 309)(%)	*P* Values
Aortic replacements				
Ascending aorta replacements	323(71.0)	117(80.1)	206(66.7)	0.003
Arch replacement				
Hemiarch replacement	15(3.3)	8(5.5)	7(2.3)	0.092
Total arch replacement	11(2.4)	6(4.1)	5(1.6)	0.115
Elephant trunk repair	4(0.9)	1(0.7)	3(1.0)	1.000
Root surgery or Aortic valve				
Aortic valve replacements	222(48.8)	77(52.7)	145(46.9)	0.247
Bentall procedure	163(35.8)	38(26.0)	125(40.5)	0.003
David procedure	17(3.7)	5(3.4)	12(3.9)	1.000
Concomitant surgery				
Any concomitant surgery	160(35.3)	47(32.2)	113(36.5)	0.361
Mitral valve replacement,	28(6.2)	5(3.4)	23(7.4)	0.142
Mitral valve repair	39(8.6)	14(9.6)	25(8.1)	0.594
Coronary artery bypass grafting	34(7.5)	5(3.4)	29(9.4)	0.023
ASD or VSD closure,	4(0.9)	2(1.4)	2(0.6)	0.597
Other	55(12.1)	21(14.4)	34(11.0)	0.355
Perfusion				
Cardiopulmonary bypass time, min	104.2 ± 40.80	101.7 ± 41.77	105.4 ± 40.34	0.362
Cross clamp time, min	64.2 ± 29.15	62.4 ± 30.78	65.1 ± 28.36	0.367
Circulatory arrest, min	1.6 ± 6.47	2.5 ± 5.96	1.2 ± 5.96	0.068
Any transfusion	304(66.8)	114(78.1)	190(61.5)	<0.001
pRBC used	261(57.4)	109(74.7)	152(49.2)	<0.001
FFP used	269(59.1)	109(74.7)	160(51.8)	<0.001
platelet used	143(31.4)	48(32.9)	95(30.7)	0.647

ASD: atrial septal defect; VSD: ventricular septal defect; PRBC, FFP, packed red blood cells; fresh frozen plasma. A *p* value < 0.05 was considered statistically significant

For the outcome of thoracic aortic surgery, both female and male patients had a lower level of aortic valve transvalvular pressure difference (female: 72.8 ± 52.62 versus 28.6 ± 12.39; *P* < 0.001; male: 52.1 ± 39.44 versus 22.4 ± 7.20; *P* = 0.008) and LV volume index (female: 41.2 ± 18.45 versus 28.7 ± 10.56; *P* < 0.001; male: 47.9 ± 21.13 versus 31.9 ± 12.97; *P* < 0.001) 7 days after surgery, indicating the early effective treatment of the surgery. There were no significant differences in the ventricular function (female: 60.2 ± 9.07 versus 58.9 ± 7.47; *P* = 0.082; male: 57.0 ± 9.49 versus 56.6 ± 9.13; *P* = 0.477) and IVST (female: 1.2 ± 0.25 versus 1.2 ± 0.19; *P* = 0.516; male: 1.3 ± 0.23 versus 1.3 ± 0.21; *P* = 0.114) between pre-operation and post-operation groups, whereas female patients had a higher LV PWT (1.2 ± 0.18 versus 1.3 ± 0.16; *P* < 0.001) after 7 days of aortic surgery (Table 4).

**Table 4:** Comparison between baseline characteristics of patients and 7-day outcomes after thoracic aortic surgery

Variable	Female (*n* = 146)		*P* value	Male (*n* = 309)		*P* value
	Pre-operation	Post-operation	*P* value	Pre-operation	Post-operation	
transvalvular pressure difference^a^	72.8 ± 52.62	28.6 ± 12.39	<0.001	52.1 ± 39.44	22.4 ± 7.20	0.008
LV volume index (ml/m2.7)^b^	41.2 ± 18.45	28.7 ± 10.56	<0.001	47.9 ± 21.13	31.9 ± 12.97	<0.001
LVEF (%)	60.2 ± 9.07	58.9 ± 7.47	0.082	57.0 ± 9.49	56.6 ± 9.13	0.477
IVST (mm)^c^	1.2 ± 0.25	1.2 ± 0.19	0.516	1.3 ± 0.23	1.3 ± 0.21	0.114
LV PWT (mm)^d^	1.2 ± 0.48	1.2 ± 0.17	0.676	1.2 ± 0.18	1.3 ± 0.16	<0.001

a: transvalvular pressure difference; b: LV volume index at end-diastole (ml/m2.7); c: IVST at end-diastole (mm); d: LV PWT at end-diastole (mm). A *p* value < 0.05 was considered statistically significant

### Primary outcomes

There was no significant dissimilarity in the mean follow-up time among women and men groups (men: 49.4 ± 14.33 mts versus Women: 49.4 ± 14.70 mts; *p* = 0.956). The overall mortality rate in the women’s groups (11.0%) was higher compared to 4.9% in the men’s groups (*p* = 0.026), as demonstrated in Table 5. The actuarial survival rate was expressively higher in male patients compared to female patients (95.1% versus 89.0% at 5 years). Neurological complications (*p* = 1.000), need for re-operation (*p* = 0.654), prolonged ventilation time (*p* = 0.204), dialysis-dependent renal failure (*p* = 1.000), ICU days (*p* = 0.229) and overall hospital stay (*p* = 0.685) were not different between two groups (Table 5).

**Table 5:** Outcome Differences Between female and male patients after thoracic aortic surgery

Variables	Overall (*n* = 455) (%)	Female (*n* = 146)(%)	Male (*n* = 309)(%)	*P* Value
In-hospital mortality	12(2.6)	5(3.4)	7(2.3)	0.534
Overall mortality	31(6.8)	16(11.0)	15(4.9)	0.026
Mean follow-up, month	49.4 ± 14.43	49.4 ± 14.70	49.4 ± 14.33	0.956
Cause of death				
Cardiac	6(1.3)	3(2.1)	3(1.0)	0.391
Aortic	6(1.3)	4(2.7)	2(0.6)	0.087
Neurological	2(0.4)	1(0.7)	1(0.3)	0.539
Cardiac re-operation	24(5.3)	9(6.2)	15(4.9)	0.654
Prolonged ventilation (> 40 h)	50(11.0)	20(13.7)	30(9.7)	0.204
Dialysis-dependent renal failure	8(1.8)	2(1.4)	6(1.9)	1.000
Neurological complications				
TIA	1(0.2)	0(0)	1(0.3)	1.000
Stroke	4(0.9)	1(0.7)	3(1.0)	1.000
Stay in ICU day	4.0 ± 5.39	4.4 ± 6.58	3.8 ± 4.73	0.229
Hospitalization, day	20.6 ± 11.43	20.3 ± 12.71	20.8 ± 10.79	0.685

TIA: transient ischemic attack. A *p* value <0.05 was considered statistically significant

Lastly, we conducted a univariate cox regression analysis of sex-specific risk factors associated with overall mortality. For male patients, several risk factors were identified, including renal failure (HR = 21.794, *p*<0.001), aortic arch contribution (HR = 13.749, *p*<0.001), and arch or/and descending aortic involvement (HR = 26.746, *p*<0.001), which had a positive impact on survival. Other risk factors that negatively influenced survival were maximum indexed aortic diameter, maximum aortic diameter, ICU stays, overall hospital stay, cardiopulmonary bypass, cross-clamp time, and circulatory arrest period. Preoperative aortic valve inadequacy positively influenced survival (HR = 0.349, *p* = 0.027) (Table 6). For female patients, NYHA I state and NYHA II had a positive impact on survival (HR = 0.068, *p* = 0.021 and HR = 0.082, *p* = 0.043, respectively), suggesting comparatively better clinical prognosis at the time of operation. In addition, maximum indexed aortic diameter and Cross clamp time were identified as risk factors associated with survival, suggesting that these patients had a higher comorbidity rate (Table 6).

**Table 6:** Gender-specific risk factors of survival in TAA patients after thoracic aortic surgery

Male	*P* value	HR (95% CI)
Maximum indexed aortic diameter	0.022	1.076(1.011–1.145)
Maximum aortic diameter	0.006	1.050(1.014–1.088)
Renal failure	<0.001	21.794(7.707–61.634)
Aortic insufficiency	0.027	0.349(0.138–0.885)
Cardiopulmonary bypass time	<0.001	1.018(1.009–1.026)
Cross clamp period	0.02	1.016(1.003–1.030)
Circulatory arrest	<0.001	1.081(1.055–1.108)
Aortic arch involvement	<0.001	13.749(5.413–34.923)
ICU stay	0.008	1.055(1.014–1.098)
Overall hospital stay	<0.001	1.034(1.016–1.052)
Arch or/and descending aortic involvement	<0.001	26.746(9.457–75.637)
**Female**	***P*** **value**	**HR (95% CI)**
Maximum indexed aortic diameter	0.027	7.357 (1.250-43.299)
Cross clamp time	0.033	1.181 (1.014–1.377)
NYHA I	0.021	0.068 (0.007–0.666)
NYHA II	0.043	0.082 (0.007–0.922)

ICU: intensive care unit; NYHA: new york heart association. A *p* value < 0.05 was considered statistically significant

## Discussion

Gender-related dissimilarities have been identified across a variety of valve, coronary, and aortic aneurysm surgeries. Thoracic aortic aneurysm (TAA) surgery remains an area where overall post-operative outcomes may be considerably improved. Complex TAA surgeries, especially hypothermic circulatory arrest (HCA), are at greater risk of surgical procedure. As such, surgical outcomes might benefit from a more individualized approach. Given the physiological and psychological differences between female and male patients, sex-related differences may exist in patients undergoing thoracic aortic surgery. In this study, we observed that women undergoing TAA surgery have a higher overall mortality rate, thus supporting gender-related differences in patients after thoracic aortic surgery.

The results of Cox regression analysis indicated that maximum indexed aortic diameter was a female-specific risk factor for overall mortality. Despite the complete diameters of the aorta being superior in men compared to women, female patients exhibited a greater diameter/BSA ratio in this study. This result was consistent with the previous study that women with smaller aorta diameters had faster disease progression because the absolute diameter was nearly equal. It suggests that the diameter of the aorta and the rate of its progression could predict the severity of the aortic diseases and thus play an important role in the formation of aortic aneurysms. Assessing sex-specific correlates of aneurysm size is important to understand and monitor the processes of poorer TAA outcomes in women as bigger aneurysms are at risk of acute aortic syndrome and are the driver of therapeutic choices [9]. These results imply differences in the mechanical pathology between women and men. It is supported by the evidence that women presented increased expression of matrix metalloproteinase 2 and 9 and decreased expression of metalloproteinase inhibitor 1 and 2 in tissues in aneurysmal disease, thus leading to increased extracellular matrix destruction in women, further resulting in greater aortic wall stiffness and pulsatile arterial load [10]. In addition, sex hormones were also reported to affect the different outcomes of abdominal aortic aneurysms. As a result, we hypothesized that a mechanical influence presented on the thoracic aortic aneurysms as indicated by another study [11]. Despite TAA being more predominant in men, women have a higher mortality rate as shown in aortic diseases [12], quicker aneurysm development, and greater dissection ratio than men. More studies are required to explore mechanisms of worse TAA consequences in women [13–17].

Additionally, both female and male patients had a lower rate of aortic valve transvalvular pressure difference and LV volume index in the early 7 days after thoracic aortic surgery, indicating the early effective treatment of the TAA surgery as shown in another study [18]. There was no significant difference in the ventricular function and interventricular septal thickness between pre-operation and post-operation (i.e. the early 7 days after surgery) groups undergoing TAAs, regardless of their gender identity which is consistent with the previous study [19]. Interestingly, preoperative aortic valve insufficiency was identified as a risk factor positively associated with survival in the male group undergoing TAAs [13–15]. In contrast, NYHA I or class II status was determined as a risk factor positively associated with survival in the female group undergoing TAAs which had been shown in mitral valve surgery [20]. These results implied that the clinical outcomes of TAA surgery were correlated with surgical timing in patients undergoing TAA surgery. In general, women receive less ideal medical and surgical treatment for diseases such as coronary artery disease, ruptured abdominal aortic aneurysms, and mitral valve disease [21]. Moreover, aortic surgery was typically implemented on females later in the illness progression – a contemporary technical problem because of smaller body size and tissue instability in women [22]. All these factors may explain worse outcomes of TAA surgery – a higher mortality rate, in female patients in comparison to male patients in the current study. Thus, more perioperative attention/care and early TAA treatment would effectively reduce the disparity of outcomes between female and male patients undergoing TAA surgery as shown in abdominal aortic aneurysm repair surgery [23]. Current guidelines are often revised through risk factor analysis, tending to decrease cut-off values for thoracic aortic surgery to 5 cm and to decrease the cut-off value for specific risk groups to 4.5 cm, such as Marfan syndrome [24].

### Limitations

There are several limitations to the current study that should be acknowledged. First, this research has been conducted at a single center, and the outcomes can not reflect practices or technologies utilized at other institutions. Second, the study was limited by its retrospective nature. Third, this study did not assess several factors such as perioperative care and drug use that may be related to gender-related outcomes. Finally, the provided samples (455 cases) in the study did not enough support the need for more statistical analysis to assess the mortality rates of different surgical approaches between male and female in TAA surgery. The subgroup analysis with the insufficient sample size would make false negative reports, therefore we abandoned subgroup analysis of different surgical approaches. Thereafter our findings would be verified in several large-scale multicenter studies.

## Conclusions

Female patients presenting with TAAs have a higher overall mortality rate and aortic diameter is associated with worse outcomes of TAA surgery. Our findings highlight sex-related differences in patients undergoing TAA surgery and thus promote the development of a personalized approach to TAA surgery with respect to sex.

## Data Availability

Data were obtained from December 2009 and December 2015 in Changhai Hospital.

## Abbreviations

TAA: Thoracic Aortic Aneurysm
LVEF: Left ventricular ejection fraction
ASD: Atrial septal defect
VSD: Ventricular septal defect
PRBC: Packed red blood cells
FFP: Fresh frozen plasma
TIA: Transient ischemic attack
